# A *Capra hircus* chromosome 19 locus linked to milk production influences mammary conformation

**DOI:** 10.1186/s40104-021-00667-y

**Published:** 2022-02-11

**Authors:** Andrew Jiang, Alex Ankersmit-Udy, Sally-Anne Turner, Megan Scholtens, Mathew D. Littlejohn, Nicolas Lopez-Villalobos, Colin G. Proser, Russell G. Snell, Klaus Lehnert

**Affiliations:** 1 Applied Translational Genetics Group, School of Biological Sciences, The University of Auckland, Auckland, New Zealand; 2 Dairy Goat Co-operative, Hamilton, New Zealand; 3 Cawthron Institute, Nelson, New Zealand; 4 AL Rae Centre of Genetics and Breeding, Massey University, Hamilton, New Zealand; 5 Dairy Cattle Breeding and Genetics, School of Agriculture & Environment, Massey University, Hamilton, New Zealand

**Keywords:** *Capra hircus*, Milk production, Pleiotropic effects, Quantitaive trait loci, Udder conformation

## Abstract

**Background:**

Economically important milk production traits including milk volume, milk fat and protein yield vary considerably across dairy goats in New Zealand. A significant portion of the variation is attributable to genetic variation. Discovery of genetic markers linked to milk production traits can be utilised to drive selection of high-performance animals.

A previously reported genome wide association study across dairy goats in New Zealand identified a quantitative trait locus (QTL) located on chromosome 19. The most significantly associated single nucleotide polymorphism (SNP) marker for this locus is located at position 26,610,610 (SNP marker rs268292132). This locus is associated with multiple milk production traits including fat, protein and volume. The predicted effect of selection for the beneficial haplotype would result in an average production increase of 2.2 kg fat, 1.9 kg protein and 73.6 kg milk yield.

An outstanding question was whether selection for the beneficial allele would co-select for any negative pleiotropic effects. An adverse relationship between milk production and udder health traits has been reported at this locus. Therefore, a genome wide association study was undertaken looking for loci associated with udder traits.

**Results:**

The QTL and production associated marker rs268292132 was identified in this study to also be associated with several goat udder traits including udder depth (UD), fore udder attachment (FUA) and rear udder attachment (RUA). Our study replicates the negative relationship between production and udder traits with the high production allele at position 19:26,610,610 (SNP marker rs268292132) associated with an adverse change in UD, FUA and RUA.

**Conclusions:**

Our study has confirmed the negative relationship between udder traits and production traits in the NZ goat population. We have found that the frequency of the high production allele is relatively high in the NZ goat population, indicating that its effect on udder conformation is not significantly detrimental on animal health. It will however be important to monitor udder conformation as the chromosome 19 locus is progressively implemented for marker assisted selection. It will also be of interest to determine if the gene underlying the production QTL has a direct effect on mammary gland morphology or whether the changes observed are a consequence of the increased milk volume.

**Supplementary Information:**

The online version contains supplementary material available at 10.1186/s40104-021-00667-y.

## Background

Genetic gain focused on production for the dairy goat population in New Zealand is limited largely to within farm selection and some buck transfers between farms. This has resulted in considerable unrealised marker driven selection potential which led to the search for and discovery of quantitative trait loci (QTL) for milk production traits in a subset of the population. In particular, the most significant QTL identified was located on chromosome 19 associated with all three production traits including fat yield, protein yield and milk volume [1]. A QTL at the same location was also reported in the French [2, 3] and British dairy goat populations [4]. A pleiotropic effect for this QTL has been described on a number of udder conformation traits including udder attachment and udder depth [3, 4]. It appears that udders in animals with higher milk production determined by the chromosome 19 locus are characterised by greater udder depth with apparent weaker and narrower udder attachment. It is important to evaluate potential negative pleiotropic effects before large scale genetic selection. There are examples where the potential introduction of alleles may have or have resulted in the introduction of negative characteristics. For example, in cattle the non-synonymous A > C mutation in a splice enhancer region of the bovine Diacylglycerol O-Acyltransferase 1 (*DGAT1*) gene dramatically reduced milk fat content but caused scouring (non-bloody watery diarrhoea), impaired growth rate and flattened intestinal microvilli [5]. Furthermore, the non-synonymous C636R mutation in bovine Mannose Receptor C Type 2 (*MRC2*) gene increased muscularity in meat breeds but caused Crooked Tail Syndrome [6, 7].

Goat udder traits and teat morphology play important roles in milk quality (somatic cell count), milking ability/ease of milking [8] and overall milk production [9]. Goat udder circumference [10, 11] and udder depth [12] have been positively correlated with milk volume. Goats with greater udder depth also had a higher somatic cell count, a negative indicator of milk quality, udder health and marker of mastitis [13]. Teat size and shape was likewise indicative of mammary gland health with shorter and narrower teats associated with a lower somatic cell count [13, 14]. These factors influence the animal’s productive lifespan or longevity [15]. Extensive selection pressure for milk production traits has been shown to exert a negative effect on udder and teat traits [3, 4, 16]. It is thereby important to consider these traits in animal selection. Udder health and conformation traits have not been assessed at scale in the New Zealand goat herd. This paper reports for the first time a genome wide investigation for goat udder traits of the New Zealand goat population using Illumina GoatSNP50 BeadChip (Illumina Inc., San Diego, CA, USA) and identifies caprine SNP marker rs268292132 as a viable selection tool for future marker assisted breeding programmes.

## Methods

### Animals and phenotypic measurements

A total of 1058 animals from 2 farms (439 animals from farm A, 619 animals from farm B) with mixed age (mean age: 4.72 years, range: 2–9 years) and of primiparous or multiparous status (mean parity: 3.72 years, range: 1–8) were scored for udder traits in February and March 2020. The New Zealand goat herd is a mixed breed population comprised of approximately 85% Saanen, 5% Toggenburg, 5% Nubian/Anglo-Nubian, 4.5% Alpine and 0.5% Sable [17]. The udder traits measured included udder depth (UD), fore udder attachment (FUA), rear udder attachment (RUA), udder furrow (UF), teat placement (TP) and teat angle (TA). Udder traits were scored on a point system from 1 to 9 described in Table 1 with definition of traits as used in McLaren et al. [18]. Additionally, the following milk production traits, milk volume, fat yield and protein yield were measured for a subset of 336 animals out of the 1058-animal cohort (141 animals from farm A, 195 animals from farm B) as part of standard herd testing procedures using Fourier transform infrared spectroscopy. Animals were of mixed age (mean age: 6.49, range: 6–9) and primiparous or multiparous status (mean parity: 5.49, range: 5–8). The animals were from the cohort described in Scholtens et al [1]. Milk production traits were modelled by extrapolation to a 305-day lactation length period, restricted to animals with lactation length > 200 days.

**Table 1:** Goat udder traits definitions

Trait	Description	Scoring system	Desired scores
Udder depth	Depth of udder measured relative to the hocks according to age	1 (deep) - 9 (shallow)	4,5,6
Rear udder attachment	Width at the top of the milk secreting tissue	1 (narrow) - 9 (wide)	9
Fore udder attachment	Attachment of udder to body wall	1 (weak) - 9 (strong)	9
Udder furrow	Strength and definition of medial suspensory ligament of udder	1 (weak) - 9 (strong)	7,8
Teat placement	Location of where teat attaches to udder	1 (wide) - 9 (close)	6,7,8
Teat angle	Teat angle from side view	1 (poor) - 9 (good)	8,9

### Genotypes

Skin samples from the 1058 phenotyped animals were collected and genotyped with the Illumina GoatSNP50 BeadChip with 53,347 SNP markers (Illumina Inc., San Diego, CA, USA) [19]. Markers with ambiguous or missing genotypes in more than 5% of goats, or with minor allele frequency < 0.01 were omitted. After filtering, 51,477 markers were retained.

### Genome-wide association analysis

Genome wide association mapping with an applied mixed linear model accounting for differences in population structure was performed using genome-wide complex trait analysis tool (GCTA) v1.93.2 [20, 21]. The following mixed linear model was used in our study:

$$ y=a+ bx+g-+e $$

*y* is the phenotype of interest, *a* is the mean term, *b* is the fixed (additive) effect of the SNP, *x* is the SNP genotype, *g*− is the effect of population structure estimated by calculating genetic relationship matrices (GRM) between all animals [21]. Mixed linear model association analysis, leave one chromosome out (MLMA-LOCO) approach was used where the chromosome on which the significant candidate SNP is located are excluded from the GRM calculations to avoid double fitting of candidate variants. *e* is the residual effect.

A SNP was considered significantly associated to a trait of interest at the whole genome level if their -log_10_ *P*-values exceeded -log_10_(5 × 10^− 8^). SNP marker trait association plots against the ARS1 goat reference [22] were constructed using the ggplot R package [23].

### Calculation of allelic effect sizes for marker rs268292132

Allelic effect sizes for marker rs268292132 were calculated for production and udder traits in the 1058 animal population using a linear regression model adjusted for age and farm origin. A one-way analysis of variance (ANOVA) test between the means (α = 0.05) was performed to characterise allelic effect differences between rs268292132 genotype groups presented in Fig. 2. The Shapiro-Wilk normality assumption test was performed on all data presented. Multiple pairwise comparisons adjusted for false discovery rate were utilised to highlight statistically significant differences in the data presented.

### Whole genome sequencing for variant identification

Whole genome sequencing was performed on 302 goats consisting of 48 animals in the cohort used for GWAS, 45 additional animals originating from farms A and B and 209 animals distributed across four other farms. The animals were selected to capture the genetic diversity of each farm. Skin samples were collected from these animals and sequenced on the Illumina HiSeq XTen platform (Illumina Inc., San Diego, CA, USA). For each animal, 700–1000 million paired end reads with read pair lengths of 150 nucleotides were generated. An average sequencing depth of 35 × was achieved. Read sequence quality was assessed using FastQC (FastQC v0.11.7). Reads were mapped to the ARS1 goat reference genome assembly using Burrows Wheeler aligner [24]. Duplicate read pairs were marked using picard MarkDuplicates (Picard v2.1.0., https://broadinstitute.github.io/picard/). Reads aligned around small insertions/deletions were realigned using the Genome Analysis Toolkit (GATK v 3.8, RealignerTargetCreator and IndelRealigner tools) [25, 26]. Probabilities for single nucleotide and indel variants were discovered individually in each alignment using GATK’s HaplotypeCaller tool [27] and genotyped jointly across the entire cohort with GATK’s GenotypeGVCFs tool. Pairwise linkage disequilibrium R^2^ values between variants were calculated using PLINK software [28]. The Ensembl Variant Effect Predictor (VEP v90.3) software (https://www.ensembl.org/vep) was used to annotate variants with their calculated variant consequences based on the ARS1 reference General Feature Format (GFF) file (http://ftp.ensembl.org/pub/release-100/gff3/capra_hircus/Capra_hircus.ARS1.100.gff3.gz).

## Results

### A chromosome 19 QTL associated with goat udder health traits in the NZ goat population

**Fig. 1:**
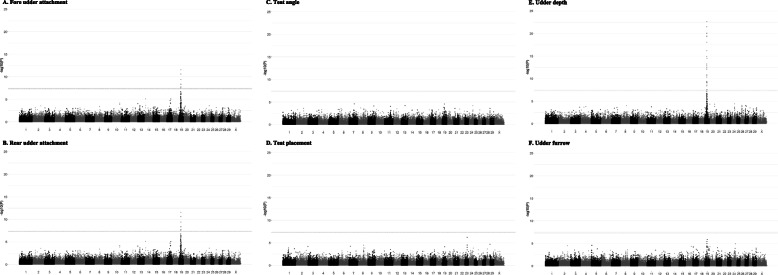
Manhattan plots of SNP marker association across chromosomes 1 to 29 plus X for Fore udder attachment (**A**), Rear udder attachment (**B**), Teat angle (**C**), Teat placement (**D**), Udder depth (**E**) and Udder furrow (**F**) for 1058 animals. Population structure was accounted for by estimating genetic relationship matrices (GRM) between all animals. Genome wide significance threshold is indicated by a dashed line at *P*-value of 5.0 × 10^−8^. SNP markers of genome wide significance are coloured in orange

We report a single genome wide significant QTL for several udder health traits including udder depth, fore udder attachment and rear udder attachment (Table 2 & Fig. 1). The QTL extend from chromosome 19 positions 24 - 29 Mb. A QTL signal was also detected for teat angle and udder furrow at the same locus but did not reach genome wide significance. No QTL association was observed for the teat placement phenotype. Fitting the most significant QTL SNP (rs268292132) as a covariate in the association analysis resulted in the ablation of the entire QTL signal, indicative of a single haplotype responsible for the signal.

**Table 2:** Genome wide significant SNPs for goat udder traits in the New Zealand goat population

Trait	SNP, Chr:Pos	dbSNP number	-log_10_(*P*-value)^1^	Annotation	Nearest gene	Distance to nearest gene, kb	MAF^2^
UD	19:25,413,768	rs268243473	8.37	Intergenic	*WSCD1*	53.48	0.352
UD	19:25,782,297	rs268243466	7.48	Intergenic	*NLRP1*	57.74	0.265
UD, RUA	19:25,823,025	rs268243465	10.53–12.07	Intergenic	*NLRP1*	40.73	0.370
UD	19:26,029,220	rs268243461	9.26	Intron	*RABEP1*	Within gene	0.249
UD, FUA, RUA	19:26,072,328	rs268243460	7.53–14.88	Intergenic	*RABEP1*	17.63	0.447
UD, RUA	19:26,115,456	rs268243459	8.30–12.83	Intergenic	*ZNF232*	23.45	0.278
UD, RUA	19:26,148,755	rs268291686	10.03–13.23	Downstream	*ZFP3*	1.30	0.409
UD, FUA, RUA	19:26,192,128	rs268243458	10.63–21.50	Downstream	*KIF1C*	3.80	0.417
UD, FUA, RUA	19:26,420,506	rs268256399	8.31–20.09	Intron	*ZMYND15*	Within gene	0.423
UD, RUA	19:26,542,254	rs268256402	9.29–12.41	Downstream	*ALOX12*	50.98	0.285
UD, FUA, RUA	19:26,578,775	rs268256403	10.58–19.47	Intergenic	*ALOX12*	14.46	0.406
UD, FUA, RUA	19:26,610,610	rs268292132	11.57–22.65	Synonymous	*RNASEK*	Within gene	0.471
UD, FUA, RUA	19:26,662,281	rs268256404	9.39–19.99	Intron	*ASGR2*	Within gene	0.454
UD	19:26,724,454	rs268256405	10.33	Intron	*DLG4*	Within gene	0.233
UD	19:26,780,952	rs268256406	9.40	Downstream	*ELP5*	0.19	0.226
UD, FUA, RUA	19:26,907,844	rs268256408	7.76–10.81	Synonymous	*NLGN2*	Within gene	0.340
UD, FUA, RUA	19:27,401,023	rs268256418	8.39–19.31	Intron	*GUCY2D*	Within gene	0.379
UD, RUA	19:27,480,793	rs268256419	12.44–18.06	Intron	*ALOXE3*	Within gene	0.418
UD, RUA	19:27,529,983	rs268256420	10.98–16.18	Intron	*VAMP2*	Within gene	0.407
UD	19:27,558,520	rs268256421	9.18	Intron	*TMEM107*	Within gene	0.308
UD	19:27,605,322	rs268256422	9.04	Intron	*CTC1*	Within gene	0.288
UD, RUA	19:27,646,998	rs268256423	7.39–10.66	Upstream	*SLC25A35*	1.18	0.254
UD, RUA	19:28,038,645	rs268256430	10.58–14.48	Intron	*PIK3R6*	Within gene	0.458
UD	19:28,202,268	rs268256435	7.57	Intergenic	*NTN1*	5.68	0.350
UD	19:28,348,471	rs268241939	7.51	Intron	*NTN1*	Within gene	0.266
UD	19:28,832,418	rs268241930	8.56	Intron	*GAS7*	Within gene	0.360
UD	19:28,953,102	rs268241927	9.13	Intron	*GAS7*	Within gene	0.270

^12^SNPs were considered significant if they exceed the genome wide significance threshold of -log_10_(5 × 10^−8^). *MAF*, Minor allele frequency; *RUA*, Rear udder attachment;* UD*, Udder depth; *FUA*, Fore udder attachment

An overlapping locus was previously identified to be associated with milk production traits in the French [3], British [4] and New Zealand goat populations [1]. The SNP most significantly associated with milk production in the New Zealand study (rs268292132) was also the most significantly associated SNP with the above udder traits in this study (Table 2 & Fig. 1).

### Whole genome sequence analysis revealed two candidate non-synonymous mutations in the proteasome 20S subunit Beta 6 (*PSMB6)* and the sex hormone binding globulin (*SHBG*) genes

Whole genome sequence analysis of 302 animals which included 48 animals in the GWAS cohort highlighted 340/40,966 variants within the 24 - 29 Mb chromosome 19 QTL interval with high linkage disequilibrium, *R*^2^ > 0.8 with the most significant SNP marker, rs268292132. Of these, no high impact variants with severe consequence on protein sequence including transcript ablation, splice acceptor, splice donor, stop gain, frameshift, stop lost and transcript amplification variants were identified (Full breakdown of the 340 variants listed in Additional file 1 & Additional file 3). Two non-synonmous mutations and one inframe deletion was predicted to alter protein sequence. The in-frame deletion in candidate gene, *SENP3* at position 27,036,323 (NC_030826.1:g. 27,036,324_27,036,326del), detected at an alternative allele frequency of 0.553 and linkage disequilibrium R^2^ value of 0.877 with the most significant SNP marker deletes a conserved glutamate (E89del). The variant has not been reported in other goat populations (Additional file 1 and Table 3). A non-synonymous variant in the candidate gene *PSMB6* at position 26,370,181 (NC_030826.1:g. 26,370,181C > T, rs647861802), observed at an alternative allele frequency of 0.566 and linkage disequilibrium R^2^ value of 0.887 with the most significant SNP marker encodes a substitution of conserved valine 221 to isoleucine (ENSCHIP00000027128.1:p.V222I, GERP conservation score = 0.9). The variant has been reported in the NextGen collaborative research project at an allele frequency of 0.038 (Additional file 1 and Table 3). A G > T transversion at position 27,087,979 in the *SHBG* gene (NC_030826.1:g. 27,087,979G > T, rs672568929) exists at an alternative allele frequency of 0.555 and linkage disequilibrium R^2^ value of 0.87 with the most significant SNP marker in the genome-sequenced animals. This variant encodes a substitution of serine 267/277/281/166 (alternative transcripts) to isoleucine (ENSCHIP00000021646.1:p.S267I/ ENSCHIP00000021662.1:p.S277I/ENSCHIP00000021669.1:p.S281I/ENSCHIP00000021683.1:p.S166I/ENSCHIP00000021685.1:p.S166I). This variant has been reported in the NextGen collaborative research project at an allele frequency of 0.028 (Additional file 1 and Table 3).

**Table 3:** Summary of candidate variants described in the study

Variant of interest	dbSNP reference number	Ref/Alt alleles	Alternative allele frequency	R^2^^1^	Effect on protein sequence	Affected gene	SIFT^2^
NC_030826.1:g. 27,036,324_27,036,326del	NA	GAG/−	0.553 (NZ)	0.877	Inframe deletion	*SENP3*	NA
NC_030826.1:g. 26,370,181C > T	rs647861802	C/T	0.566 (NZ) 0.038 (NextGen)	0.887	Missense mutation	*PSMB6*	0.33
NC_030826.1:g. 27,087,979G > T	rs672568929	G/T	0.555 (NZ) 0.028 (NextGen)	0.870	Missense mutation	*SHBG*	0.01

^12^Linkage disequilibrium values were calculated between the variant of interest and the most significant QTL marker rs268292132 (chr19:26,610,610). SIFT predicts whether an amino acid substitution affects protein function based on sequence homology and the amino acid properties. SIFT scores range from 0.0 (deleterious) to 1.0 (tolerated)

### An adverse relationship between milk production traits and udder conformation

**Fig. 2:**
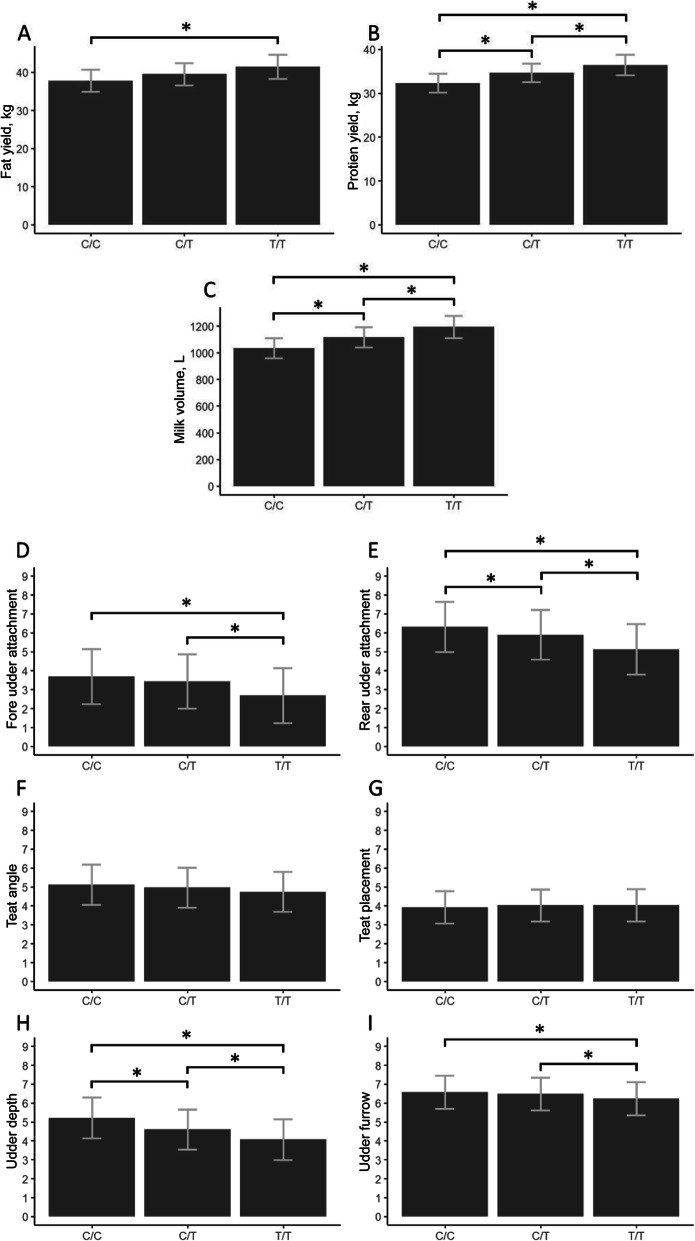
Allelic effect sizes for marker rs268292132 genotype groups. Production traits, Milk Fat (**A**), Milk Protein (**B**), Milk volume (**C**) and udder traits, Fore udder attachment (**D**), Rear Udder Attachment (**E**), Teat Angle (**F**), Teat Placement (**G**), Udder Depth (**H**), Udder Furrow (**I**) were plotted as least squared means with 95% confidence intervals. Asterisks indicate pairwise significant differences between genotype groups (*P*-value < 0.5, multiple pairwise comparisons adjusted for false discovery rate; α = 0.05)

Allelic effect sizes for marker rs268292132 adjusted for age and farm origin were calculated in our GWAS population. An average difference of 3.66 kg fat (*P*-value = 3.09 × 10^− 4^), 4.12 kg protein (*P*-value = 2.58 × 10^− 8^) and 159.52 L volume (*P*-value = 7.22 × 10^− 10^) was recorded when comparing opposing homozygotes (Fig. 2 & Table 4). The production gains were accompanied by a decrease of 1.14 score points in udder depth (*P*-value = 9.31 × 10^− 18^), 0.99 score points in fore udder attachment (*P*-value = 5.05 × 10^− 7^) and 1.19 score points in rear udder attachment (*P*-value = 1.08 × 10^− 12^) when selecting for the high production T allele (Fig. 2 & Table 4).

**Table 4:** Allelic effect sizes for rs268292132 SNP marker (chr19:26,610,610)

Phenotypic trait	rs268292132 marker genotype		
	CC	CT	TT
*Milk production traits*			
Milk fat (kg) ^1^	37.78 (34.87–40.69)	39.55 (36.64–42.47)	41.44 (38.25–44.63)
Milk protein (kg) ^1^	32.31 (30.16–34.46)	34.66 (32.52–36.81)	36.43 (34.08–38.78)
Milk volume (L)^1^	1033.76 (958.75–1108.78)	1116.52 (1041.5–1191.54)	1193.28 (1111.12–1275.44)
*Udder traits*			
Fore udder attachment^1^	3.69 (2.24–5.13)	3.44 (2.01–4.87)	2.70 (1.25–4.14)
Rear udder attachment^1^	6.32 (4.99–7.64)	5.89 (4.58–7.20)	5.13 (3.80–6.46)
Teat angle	5.11 (4.05–6.18)	4.96 (3.91–6.01)	4.75 (3.68–5.81)
Teat placement	3.92 (3.07–4.77)	4.03 (3.18–4.87)	4.03 (3.18–4.88)
Udder depth^1^	5.21 (4.13–6.29)	4.60 (3.53–5.66)	4.07 (2.99–5.15)
Udder furrow	6.57 (5.70–7.45)	6.48 (5.61–7.34)	6.23 (5.36–7.11)

Phenotypic values were taken as averages of animals (95% confidence intervals in brackets) with each corresponding genotype. Values were adjusted for birth year and farm origin.^1^Traits associated with marker rs268292132

## Discussion

A genome wide association study with an applied mixed model to account for differences in population structure was performed in 1058 dairy goats in New Zealand. The study highlighted a QTL located on chromosome 19 (24–29 Mb) associated with udder depth, fore udder attachment and rear udder attachment. This QTL was also identified in a previous study of the dairy goat population in New Zealand to be associated with milk production traits; fat yield, protein yield and milk volume (as described in Scholtens et al. [1]). Our findings are in agreement with trait effects of an overlapping QTL previously reported in French dairy goats for milk fat content, milk protein content, udder depth and rear udder attachment [2, 3] and British dairy goats for milk volume, udder depth and udder attachment [4]. The pleiotropic effect of this locus on both production and udder phenotypes combined with a high density of genes within the region (22.5 genes/Mb) has made it difficult to nominate candidate genes for the observed traits. Martin and colleagues suggested several fatty acid and lipid metabolism pathway genes including phospholipase D2 *(PLD2*), gamma-glutamyltransferase 6 (*GGT6*) and the arachiodonate lipoxygenase (*ALOX*) family of genes [3]. Mucha and colleagues also noted a potential role for protein stability and lipid homeostasis gene, asialoglycoprotein receptor 2 (*ASGR2*) in udder formation [4]. Our analysis from 302 whole genome sequenced animals showed no protein-altering variants for *PLD2*, *GGT6*, *ASGR1/ASGR2* and *ALOX* genes.

Whole genome sequence analysis revealed 340 variants within the 5 Mb QTL with linkage disequilibrium *R*^2^ > 0.8 with the most significant SNP marker rs268292132. After removing markers not predicted to change protein sequence, two non-synonmous variants and one inframe deletion variant were of interest. We propose two candidate non-synonymous variants including a valine to isoleucine variant (V222I) in the proteasome 20S subunit 6 (*PSM6B*) gene, and a serine to isoleucine variant (S267I/S277I/S281I/S166I) in the sex hormone binding globulin (*SHBG*) gene. SIFT scores were predicted at 0.3 and 0.01 respectively suggesting a likely deleterious effect for the *SHBG* mutation. Genetic variation in human PSM6B was associated with LDL cholesterol and total cholesterol levels in a meta-analysis of 617,303 individuals of multi-ethnic backgrounds with 32 million genotyped and imputed variants [29]. *PSMB6* encodes a core component of the 20S proteasome complex involved in intracellular protein degradation. However, its role in lipid metabolism is unclear. In the same GWAS study, genetic variants in *SHBG* were associated with triglyceride levels [29]. *SHBG* regulates the plasma metabolic clearance rate of steroid hormones such as oestrogen [30–32], although like *PSMB6* a strong functional link with lipid metabolism is not yet determined. Additionally, a GAG glutamate inframe deletion (E89del) was detected in the candidate gene Sentrin-specific protease 3 (*SENP3*). Limited knowledge exists in the literature on the mechanistic role of *SENP3* in milk production or morphological udder development. Given its widespread roles in protein stability, chromatin organisation, transcription, DNA repair, autophagy, protein localisation and homeostasis, a number of potential mechanisms maybe of interest [33]. Attributing causality to either of these mutations will require further genotyping of these variants within a study population that contain sufficient meioses to separate out causative haplotypes and appropriate functionality testing. Despite directing the majority of our focus to variants predicted to impact protein sequence as a direct link to functionality, we acknowledge that non-coding and regulatory variants are also of interest. Indeed our whole genome analysis have revealed two splice region variants, one 5′ UTR variant and five 3′ UTR variants within the QTL that exists in high linkage disequilibrium with the most significant QTL SNP marker. Future studies involving these variants in eQTL analyses or chromatin accessibility analyses such as ChIP-seq or ATAC-seq or experimental CRISPR-cas9 variant models could provide additional insight into biological causality. Moreover, future investigation and characterisation of large copy number variants in our dataset could additionally reveal candidate variants not detectable by SNP chip methods.

Selection on the chromosome 19 QTL for milk production traits will hold economic value in dairy industries. Markers capturing the QTL effect may be utilised in marker-assisted breeding programmes to drive selection of the next generation of high-performance animals. Employing selection with the most significant marker, rs268292132 (chr19:26,610,610) was predicted to increase fat yield, protein yield and milk volume in our study population. However, a potential adverse relationship between production traits and udder conformation has been indicated for this locus [3, 4]. Our study replicated the observations of a shift in the negative direction for udder depth, fore udder attachment and rear udder attachment scores. It is unclear whether the negative correlation between production and udder traits are a consequence of increased milk production and udder volume retention giving rise to a perceived increase in udder depth with weak and narrow udder attachment, or a true biological deterioration of udder morphology independent of the production increase. In the future, it will be interesting to determine whether the gene underlying the QTL has a direct effect on mammary gland morphology. Both alleles at the rs268292132 locus are common in the dairy goat population, with the high-milk production allele approaching fixation on some farms after long-term phenotypic selection for both milk production and conformation traits. These observations imply that the magnitude of the udder effects may be well-balanced with the increase in milk production. A comprehensive analysis of udder shape deterioration versus the production increase across the wider population will be required to optimise selection and breeding programmes for marker rs268292132 as these traits affect production [9], milk quality (somatic cell count) [13], milking ability [8] and total number of days in production [15].

The overarching goal of this research is development of a marker-assisted breeding programme that can be implemented to accelerate productivity gain. This study identified use of the rs268292132 SNP as a viable selection marker for marker assisted breeding programmes to increase production.

## Conclusion

Genome wide association analyses across the dairy goat population in New Zealand have discovered a 24-29 Mb QTL on caprine chromosome 19 that has a pleiotropic effect on milk production and udder conformation traits. A negative correlation between production and udder conformation traits was observed in the New Zealand goat population which corroborates reports in French and British dairy goat herds. Close monitoring of udder health is recommended for future selection programmes involving this locus. Marker assisted breeding programmes with markers effectively identifying this QTL can be utilised to select the next generation of high-performance animals to accelerate productivity gain.

## Supplementary Information

**Additional file 1 Supplementary 1** Full protein sequence alignment between species across several taxa indicating the degree of conservation around the *SENP3* E89del (A), *PSMB6* V222I (B) and *SHBG* S267I/S277I/S281I/S166I (C) variant sites (outlined in red). Multiple sequence alignment was performed with ClustalW. NCBI or UniProt accession numbers provided in brackets. Sequence logos were created using WebLogo web based application [34]

**Additional file 2 Supplementary 2** Summary of the 340 whole genome sequence variants identified from 302 goats located within the chromosome 19 QTL interval with high linkage disequilibrium, *R*^2^ > 0.8 with the most significant SNP marker rs268292132

**Additional file 3 Supplementary 3** Ensembl Variant Effect Predictor (VEP) calculated variant consequences on the 340 variants identified from whole genome sequencing of 302 goats

## Data Availability

Supporting data is available through contact with the authors.

## Abbreviations

QTL: Quantitative trait loci
eQTL: Expression quantitative trait loci
SNP: Single nucleotide polymorphism
GWAS: Genome wide association study
UD: Udder depth
FUA: Fore udder attachment
RUA: Rear udder attachment
UF: Udder furrow
TP: Teat placement
TA: Teat angle
*DGAT1*: Diacylglycerol o-acyltransferase 1
*MRC2*: Mannose receptor C type 2
GCTA: Genome-wide complex trait analysis tool
GRM: Genetic relationship matrices
MLMA-LOCO: Mixed linear model association analysis, leave one chromosome out
ANOVA: Analysis of variance
*PSMB6*: Proteasome 20S subunit beta 6
*SHBG*: Sex hormone binding globulin
*PLD2*: Phospholipase D2
*GGT6*: Gamma-glutamyltransferase 6
*ALOX*: Arachiodonate lipoxygenase
*ASGR2*: Asialoglycoprotein receptor 2
*SENP3*: Sentrin-specific protease 3
ChIP-seq: Chromatin immunoprecipitation sequencing
CRISPR-Cas9: Clustered regularly interspaced short palindromic repeats-CRISPR-associated protein 9
ATAC-seq: Assay for transposase-accessible chromatin using sequencing.
