# Supplementary figures and images for: A Capra hircus chromosome 19 locus linked to milk production influences mammary conformation
Source: J Anim Sci Biotechnol. 2022 Feb 11;13:4. doi: 10.1186/s40104-021-00667-y (PMC8832686; doi:10.1186/s40104-021-00667-y)

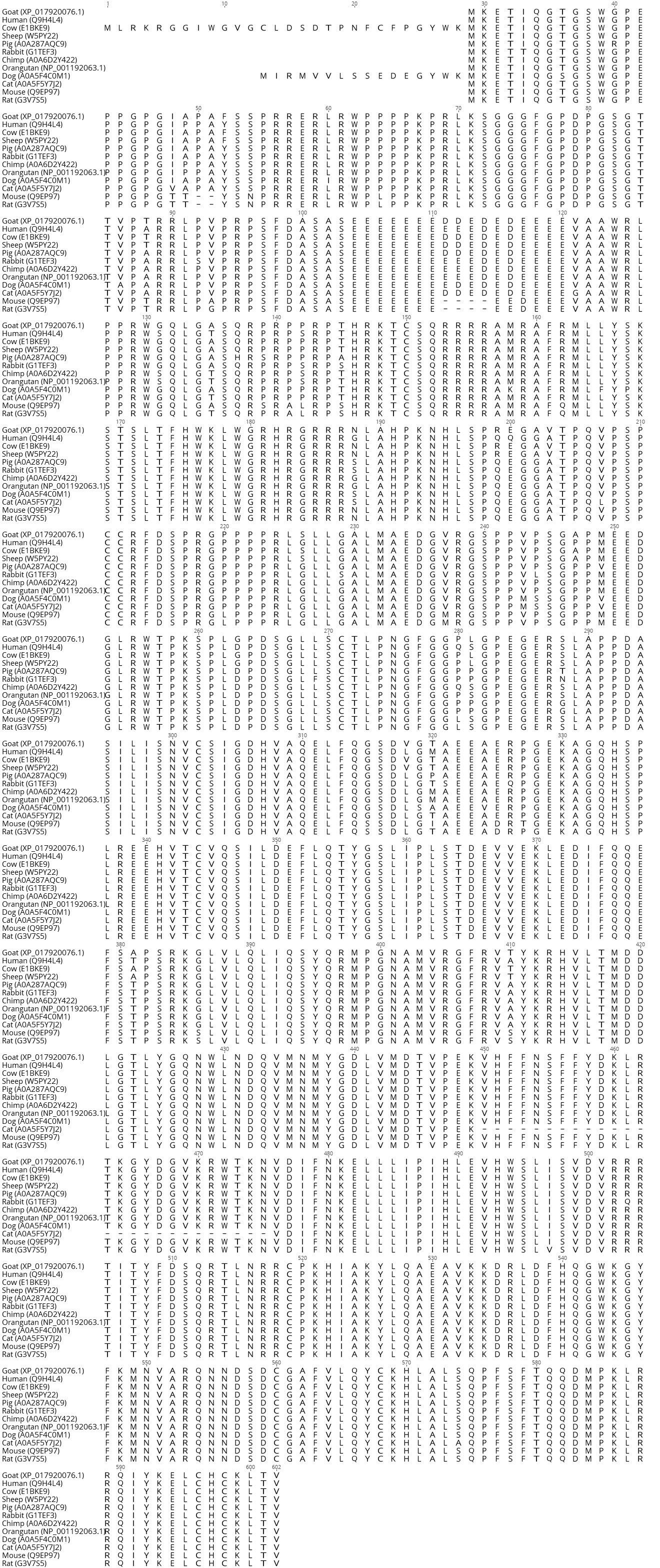

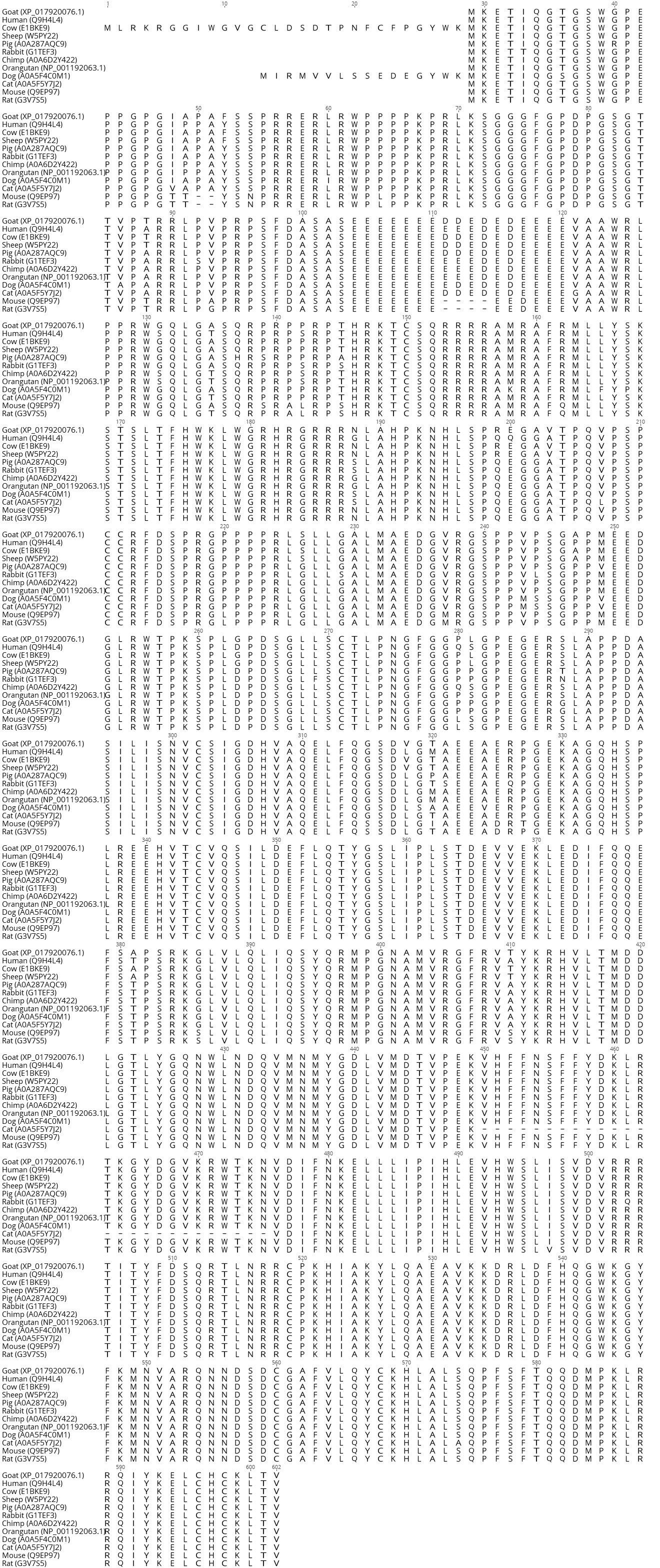
**A** SENP3

**E89del**


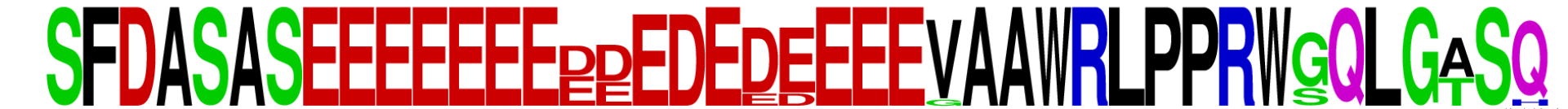

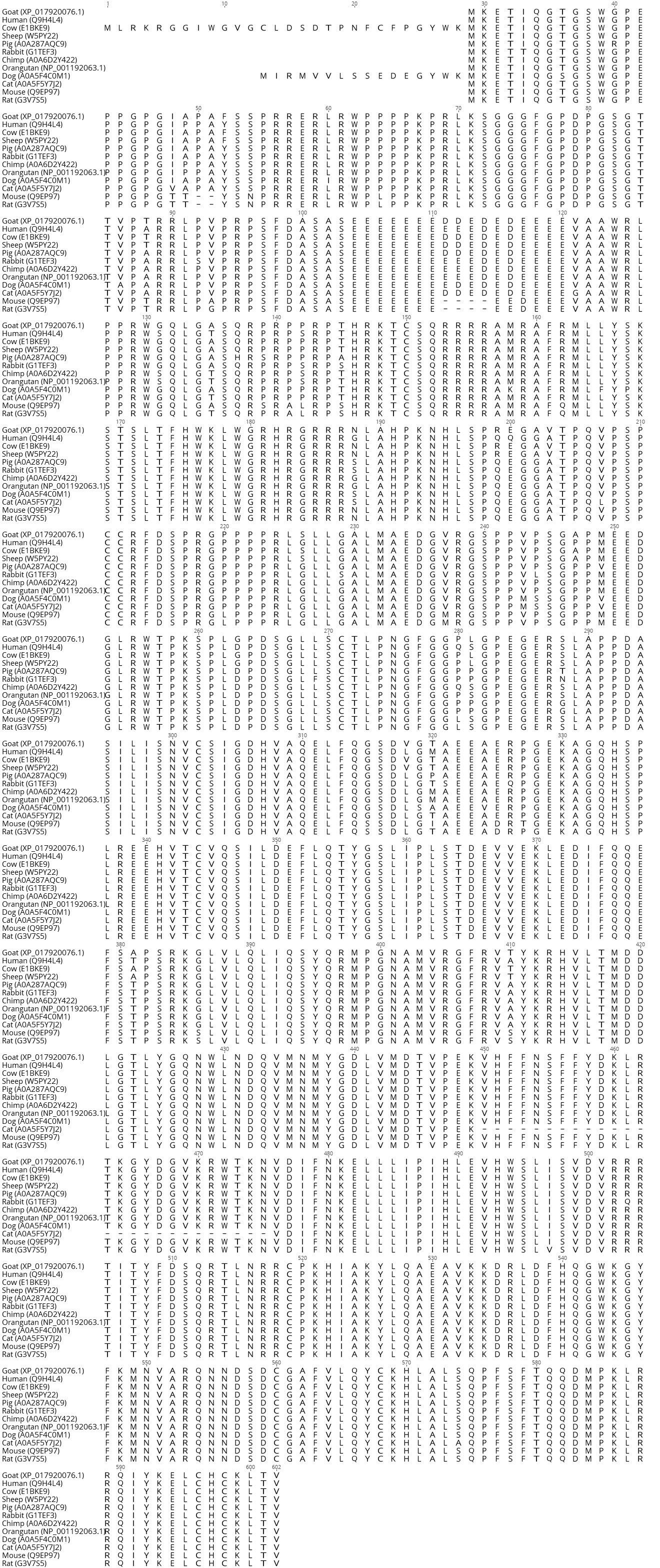


**E89del**


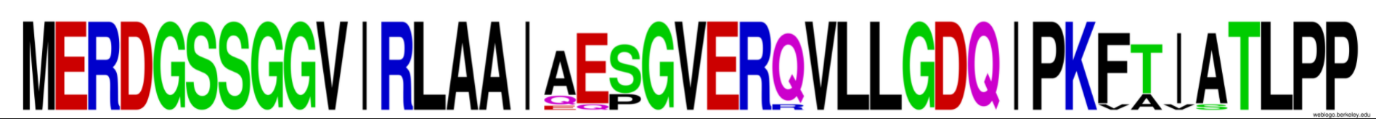

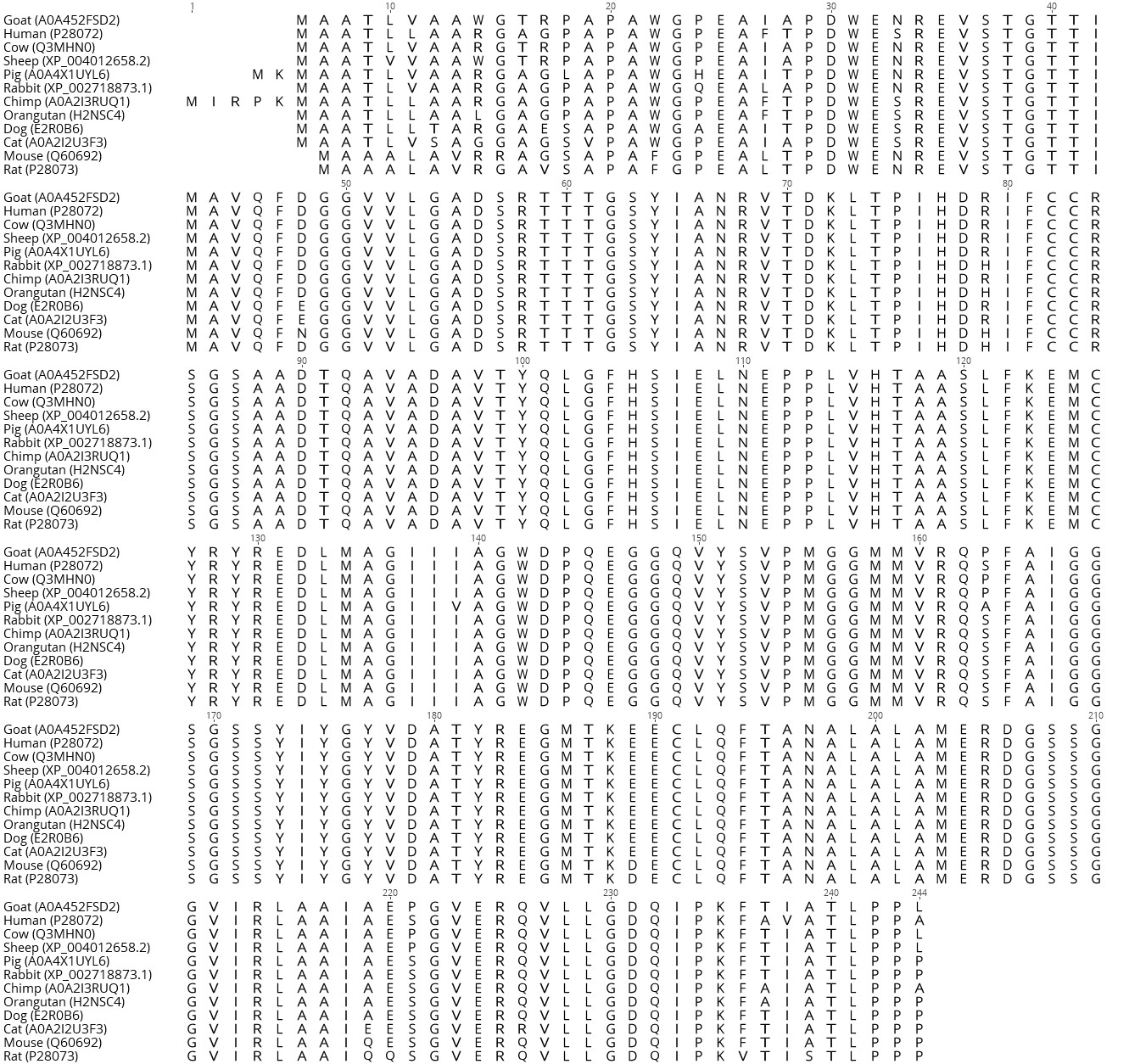
**B** PSMB6

**V222**

**V222**


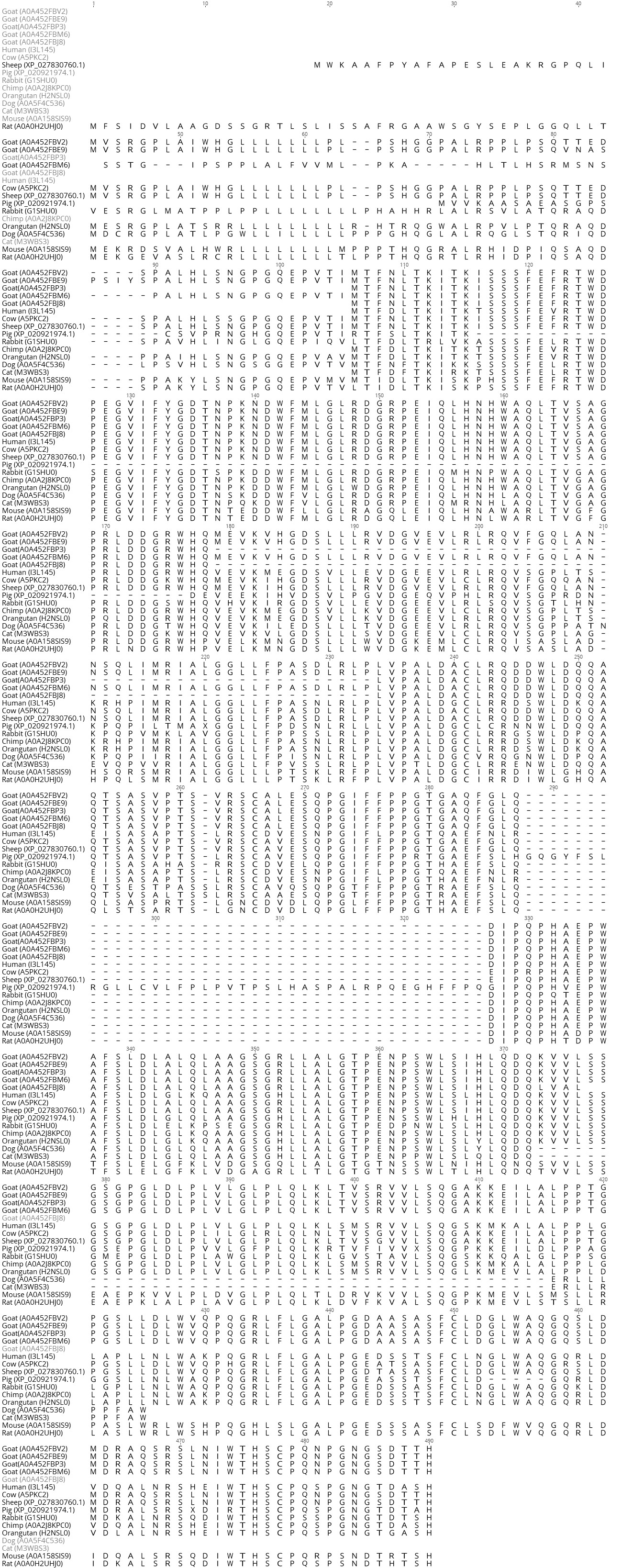
**C** SHBG


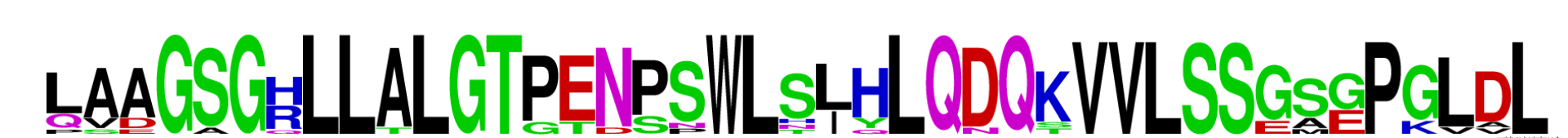

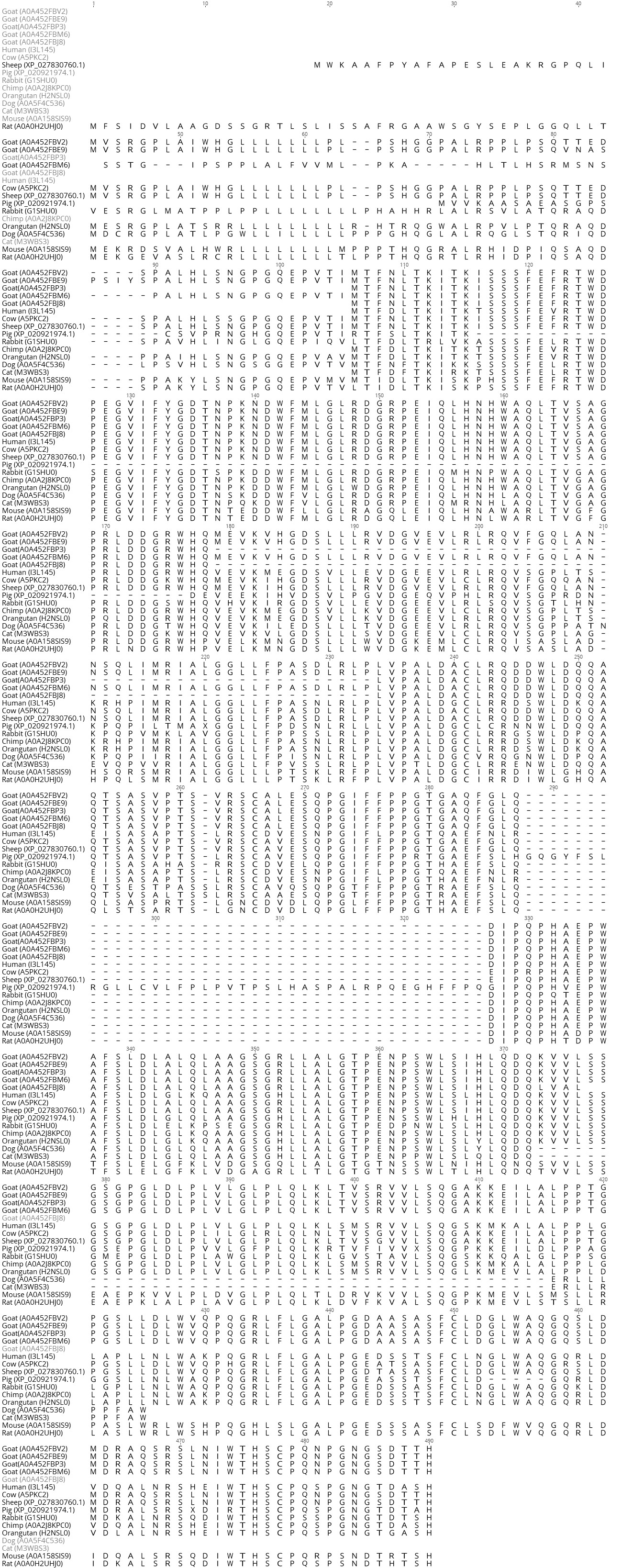

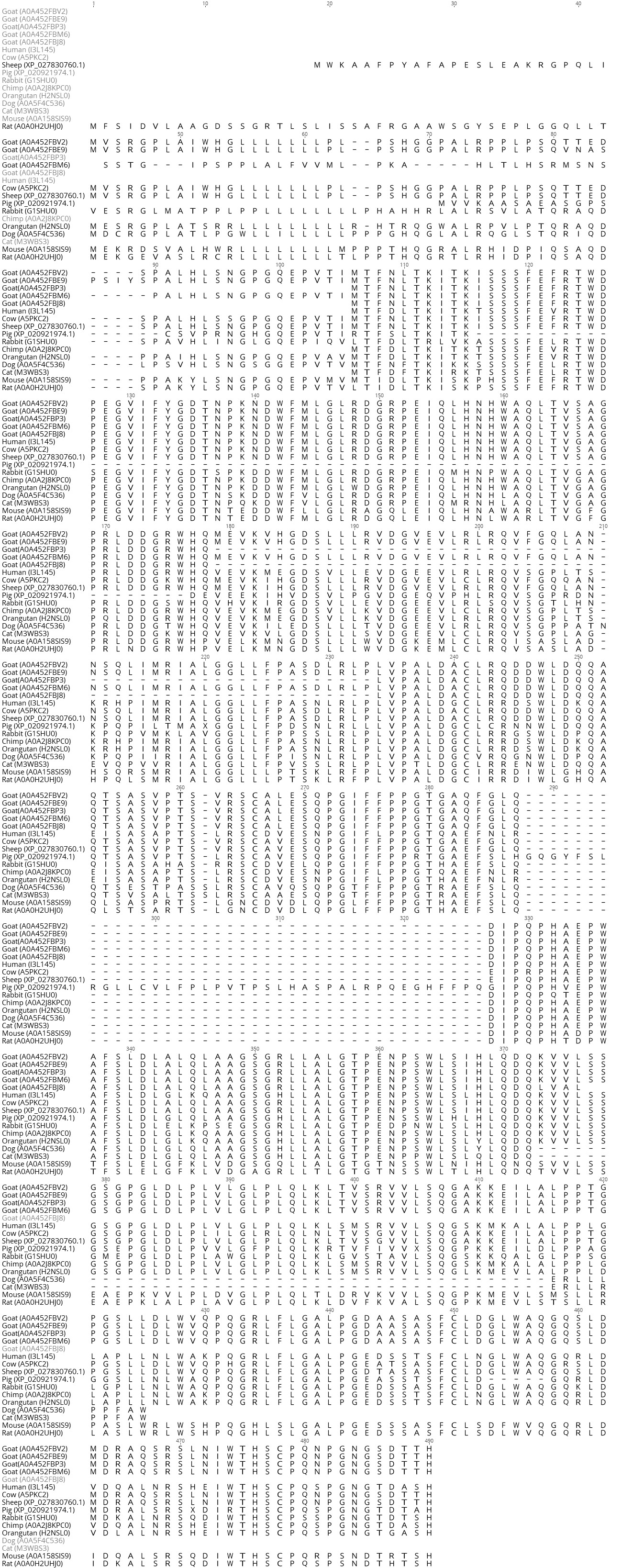


**S267/S277/S281/S166**

**S267/S277/S281/S166**

Supplement: Supplementary file 1 — Additional file 1 Supplementary 1 Full protein sequence alignment between species across several taxa indicating the degree of conservation around the SENP3 E89del (A), PSMB6 V222I (B) and SHBG S267I/S277I/S281I/S166I (C) variant sites (outlined in red). Multiple sequence alignment was performed with ClustalW. NCBI or UniProt accession numbers provided in brackets. Sequence logos were created using WebLogo web based application [34] [file 40104_2021_667_MOESM1_ESM.docx]
