# Supplementary material for: A Capra hircus chromosome 19 locus linked to milk production influences mammary conformation
Source: J Anim Sci Biotechnol. 2022 Feb 11;13:4. doi: 10.1186/s40104-021-00667-y (PMC8832686; doi:10.1186/s40104-021-00667-y)
Supplement: Supplementary file 3 — Additional file 3 Supplementary 3 Ensembl Variant Effect Predictor (VEP) calculated variant consequences on the 340 variants identified from whole genome sequencing of 302 goats [file 40104_2021_667_MOESM3_ESM.docx]

| Sequence Ontology | Description | Impact on protein sequence | Variants assigned |
| --- | --- | --- | --- |
| Transcript ablation | A feature ablation whereby the deleted region includes a transcript feature | HIGH | 0 |
| Splice acceptor variant | A splice variant that changes the 2-base region at the 3' end of an intron | HIGH | 0 |
| Splice donor variant | A splice variant that changes the 2-base region at the 5' end of an intron | HIGH | 0 |
| Stop gained | A sequence variant whereby at least one base of a codon is changed, resulting in a premature stop codon, leading to a shortened transcript | HIGH | 0 |
| Frameshift variant | A sequence variant which causes a disruption of the translational reading frame, because the number of nucleotides inserted or deleted is not a multiple of three | HIGH | 0 |
| Stop lost | A sequence variant where at least one base of the terminator codon (stop) is changed, resulting in an elongated transcript | HIGH | 0 |
| Start lost | A codon variant that changes at least one base of the canonical start codon | HIGH | 0 |
| Transcript amplification | A feature amplification of a region containing a transcript | HIGH | 0 |
| Inframe insertion | An inframe non-synonymous variant that inserts bases into in the coding sequence | MODERATE | 0 |
| Inframe deletion | An inframe non-synonymous variant that deletes bases from the coding sequence | MODERATE | 1 |
| Missense variant | A sequence variant, that changes one or more bases, resulting in a different amino acid sequence but where the length is preserved | MODERATE | 2 |
| Protein altering variant | A sequence variant which is predicted to change the protein encoded in the coding sequence | MODERATE | 0 |
| Splice region variant | A sequence variant in which a change has occurred within the region of the splice site, either within 1-3 bases of the exon or 3-8 bases of the intron | LOW | 2 |
| Incomplete terminal codon variant | A sequence variant where at least one base of the final codon of an incompletely annotated transcript is changed | LOW | 0 |
| Start retained variant | A sequence variant where at least one base in the start codon is changed, but the start remains | LOW | 0 |
| Stop retained variant | A sequence variant where at least one base in the terminator codon is changed, but the terminator remains | LOW | 0 |
| Synonymous variant | A sequence variant where there is no resulting change to the encoded amino acid | LOW | 10 |
| Coding sequence variant | A sequence variant that changes the coding sequence | MODIFIER | 0 |
| Mature miRNA variant | A transcript variant located with the sequence of the mature miRNA | MODIFIER | 0 |
| 5’ prime UTR variant | A UTR variant of the 5' UTR | MODIFIER | 1 |
| 3’ prime UTR variant | A UTR variant of the 3' UTR | MODIFIER | 5 |
| Non-coding transcript exon variant | A sequence variant that changes non-coding exon sequence in a non-coding transcript | MODIFIER | 0 |
| Intron variant | A transcript variant occurring within an intron | MODIFIER | 133 |
| NMD transcript variant | A variant in a transcript that is the target of NMD | MODIFIER | 0 |
| Non-coding transcript variant | A transcript variant of a non coding RNA gene | MODIFIER | 0 |
| Upstream gene variant | A sequence variant located 5' of a gene | MODIFIER | 116 |
| Downstream gene variant | A sequence variant located 3' of a gene | MODIFIER | 124 |
| TFBS ablation | A feature ablation whereby the deleted region includes a transcription factor binding site | MODIFIER | 0 |
| TFBS amplification | A feature amplification of a region containing a transcription factor binding site | MODIFIER | 0 |
| TF binding site variant | A sequence variant located within a transcription factor binding site | MODIFIER | 0 |
| Regulatory region ablation | A feature ablation whereby the deleted region includes a regulatory region | MODERATE | 0 |
| Regulatory region amplification | A feature amplification of a region containing a regulatory region | MODIFIER | 0 |
| Feature elongation | A sequence variant that causes the extension of a genomic feature, with regard to the reference sequence | MODIFIER | 0 |
| Regulatory region variant | A sequence variant located within a regulatory region | MODIFIER | 0 |
| Feature truncation | A sequence variant that causes the reduction of a genomic feature, with regard to the reference sequence | MODIFIER | 0 |
| Intergenic variant | A sequence variant located in the intergenic region, between genes | MODIFIER | 129 |
